# Genomic and Phenotypic Udder Evaluation for Dairy Cattle Selection: A Review

**DOI:** 10.3390/ani13101588

**Published:** 2023-05-09

**Authors:** Miguel A. Gutiérrez-Reinoso, Pedro M. Aponte, Manuel García-Herreros

**Affiliations:** 1 Carrera de Medicina Veterinaria, Facultad de Ciencias Agropecuarias y Recursos Naturales, Universidad Técnica de Cotopaxi (UTC), Latacunga 0501491, Ecuador; miguel.gutierrez@utc.edu.ec; 2 Laboratorio de Biotecnología Animal, Departamento de Ciencia Animal, Facultad de Ciencias Veterinarias, Universidad de Concepción (UdeC), Chillán 3780000, Chile; 3 Colegio de Ciencias Biológicas y Ambientales (COCIBA), Universidad San Francisco de Quito USFQ, Quito 170157, Ecuador; pmaponte@usfq.edu.ec; 4 Colegio de Ciencias de la Salud, Escuela de Medicina Veterinaria, Universidad San Francisco de Quito USFQ, Quito 170157, Ecuador; 5 Campus Cumbayá, Instituto de Investigaciones en Biomedicina “One-Health”, Universidad San Francisco de Quito USFQ, Quito 170157, Ecuador; 6 Instituto Nacional de Investigação Agrária e Veterinária (INIAV), 2005-048 Santarém, Portugal

**Keywords:** genomic analysis, phenotypic traits, evaluation criteria, milk production, health, welfare, longevity, dairy cattle

## Abstract

**Simple Summary:**

Genomic and phenotypic selection criteria have been crucial in dairy cattle during the last decade. Udder health and milk production are important factors affecting productivity in dairy cattle. Furthermore, genomic and phenotypic selection are essential tools for increasing milk supply for human consumption, decreasing the use of antimicrobial products, improving animal health and welfare, and developing efficient dairy cattle production systems. The main aim of the present review is to explore the current advances, novel strategies, and future challenges in genomic and phenotypic udder evaluation traits for dairy cattle selection. Thus, the present review will explain the importance of genomic and phenotypic characterization of udder traits related to dairy cattle’s health, production, and longevity. This review will encompass a comprehensive overview of the criteria for genomic and phenotypic udder evaluation and highlight potential future perspectives.

**Abstract:**

The traditional point of view regarding dairy cattle selection has been challenged by recent genomic studies indicating that livestock productivity prediction can be redefined based on the evaluation of genomic and phenotypic data. Several studies that included different genomic-derived traits only indicated that interactions among them or even with conventional phenotypic evaluation criteria require further elucidation. Unfortunately, certain genomic and phenotypic-derived traits have been shown to be secondary factors influencing dairy production. Thus, these factors, as well as evaluation criteria, need to be defined. Owing to the variety of genomic and phenotypic udder-derived traits which may affect the modern dairy cow functionality and conformation, a definition of currently important traits in the broad sense is indicated. This is essential for cattle productivity and dairy sustainability. The main objective of the present review is to elucidate the possible relationships among genomic and phenotypic udder evaluation characteristics to define the most relevant traits related to selection for function and conformation in dairy cattle. This review aims to examine the potential impact of various udder-related evaluation criteria on dairy cattle productivity and explore how to mitigate the adverse effects of compromised udder conformation and functionality. Specifically, we will consider the implications for udder health, welfare, longevity, and production-derived traits. Subsequently, we will address several concerns covering the application of genomic and phenotypic evaluation criteria with emphasis on udder-related traits in dairy cattle selection as well as its evolution from origins to the present and future prospects.

## 1. Introduction

For decades, dairy cattle breeding has been focused on increasing milk production [1]. Many functional traits have been described as having negative genomic correlations with milk production as well as reductions in genetic merit for health and fitness [2]. However, one of the constant goals and challenges in high-producing dairy cattle has been to balance fertility, udder health, and metabolic diseases without affecting milk production or compromising animal welfare [1,2,3,4]. 

Genomic and phenotypic evaluation methods provide accurate and efficient evaluations of udder traits in dairy cattle, allowing farmers to make more informed breeding decisions for animal selection. Phenotypic evaluation methods use physical measurements and observations to evaluate udder traits, providing a more holistic evaluation of the cow’s overall health and productivity. On the other hand, genomic evaluation methods use DNA sequencing to identify specific genetic markers associated with desirable udder traits, providing a more direct and precise evaluation. 

Combining genomic and phenotypic evaluation methods can provide a complete picture of a cow’s udder health and milk production potential, allowing for more effective breeding and selection decisions. Furthermore, using genomic and phenotypic evaluation methods can ultimately lead to improved udder health, milk production, and overall profitability for dairy farmers and enhanced quality of dairy products for consumers.

In the dairy cow, different factors, such as udder conformation and size and environmental conditions, contribute to variations in cow performance and functionality, mainly in longevity and productivity [5]. In recent years, dairy cattle selection has been based on female-derived traits such as milk production performance and linear conformation traits [1]. Several of these linear conformation traits are negatively correlated with other functional traits, such as milk production, which has led to a reduction in health, body condition, and adaptability-derived traits [6]. Over the last 10–20 years, other traits related to udder health, such as somatic cell counts (SCCs), productive life (longevity), and other reproductive-related traits, such as daughter fertility, have been incorporated [3]. These traits have become increasingly important in the dairy industry, and recently, genomic selection has created new opportunities for increased accuracy of the selection of such traits in very young animals [3,7]. In other ruminant species, highly efficient selection schemes were established based on pyramidal population management with core breeders at the top. The selection schemes were based on pedigree and official milk records, artificial insemination results, controlled mating, and the estimation of breeding values with the aim of accelerating genetic progress [1]. On the other hand, the constant participation of young bulls in testing and breeding programs has been described in different dairy herds in the U.S. [8]. For bull selection, udder conformation and the expected difference in milk production were considered the most essential traits. However, traditionally, the most important traits for cow selection were milk production followed by udder conformation, feet and legs, and fat percentage [8]. Nowadays, the need to preserve particular traits of economic interest for milk production and to ensure positive genetic progress is one of the main objectives [9,10,11]. Therefore, current selection programs require specific breeding plans that take into account milk production and functional traits, including those related to udder conformation and functionality [12].

Moreover, the heritability assessment and genetic correlations between traits and selection indices allow identifying the most suitable individuals for selection in dairy breeds [11,13]. Poor udder and teat conformation has been reported to reduce profitability in dairy herds [14]. Furthermore, this has an impact on the incidence of mastitis at calving and leads to decreased productivity during the lifetime of the cow [9]. Therefore, it is necessary to evaluate the effects of udder conformation on performance and future progeny. 

The objective of the present review is to provide a comprehensive description of the influence of genomic and phenotypic evaluation criteria in dairy cattle selection. It also aims to answer how these criteria may alleviate the adverse effects of defective udder conformation and function on different traits related to udder health, longevity, and milk production. Subsequently, we will address several concerns covering the application of genomic and phenotypic evaluation criteria in dairy cattle, its evolution from origins to the present day, and future prospects. This review is designed and organized as follows: The first part will briefly describe the udder evaluation criteria from the past to the present. In the second part, the use of the evaluation criteria based on udder-derived genomic and phenotypic traits as predictive tools for dairy cattle selection will be described, as well as several methodologies used for their application. In the third part, genomic and phenotypic evaluation criteria as evaluation tools for different traits associated with udder health, milk production (yield and quality), linear conformation, and longevity will be covered. Moreover, this part includes highly predictive genomic and phenotypic-derived methods that are key to the prognosis of other complex traits which directly or indirectly influence the udder conformation and its functionality. Incorporating both genomic and phenotypic evaluation criteria shows great promise in enhancing the accuracy of predicting udder-related traits and improving dairy cattle selection. However, this approach also presents several challenges, including integrating and optimizing these evaluation methods to develop the ideal selection process for enhancing udder conformation and functionality. Maximizing genetic gains across multiple traits associated with udder health, longevity, and milk production in dairy cattle has the potential to significantly boost livestock productivity and profitability in the future. By prioritizing these traits in breeding programs, farmers can improve the overall health and milk output of their herds, resulting in higher profits per animal and a more sustainable and efficient dairy industry.

## 2. Udder Evaluation Criteria: Past and Present

Udder evaluation has been a part of dairy cattle selection for centuries, with early breeders selecting cows based on visual inspection and manual palpation of the udder. In the early 1900s, standardized methods for udder evaluation were developed, including the linear score system and the udder index score. Dairy cattle breed associations have been developing classification systems since 1929, when the Holstein-Friesian Association of America introduced the first official system, intending to utilize these systems as phenotypic or genetic indicators of milk production ability, longevity, and udder health and milk production capacity over time [2,3,4,5,7,8,9].

In the mid-1900s, milk production testing became a common method for evaluating udder health and milk quality, with farmers tracking the amount and quality of milk produced by each cow. The establishment of modern livestock breeding programs dates back to the 1960s, when nucleus breeding initiatives emerged that utilized official pedigree data and milk production records to drive genetic progress [3]. This approach allowed registered dairy herds to make significant strides in genetic improvement, which could then be effectively disseminated to commercial herds [1]. As a result, these breeding programs have played a critical role in transforming the dairy industry and driving improvements in milk yield, udder health, and other key traits that impact profitability and sustainability. 

While increasing milk production and improving quality (such as fat and protein content) continues to be the top priority for profitability, livestock breeders have also begun considering other important traits for selection. These may include mechanical milking ability, udder health, disease resistance (e.g., against mastitis and internal parasites), and even the nutritional value of milk (such as fatty acid composition) [1,15]. Given the importance of considering a range of traits beyond just milk production and quality, the dairy industry has increasingly focused on developing comprehensive selection criteria that consider factors such as udder health, disease resistance, and mechanical milking ability. One example of this is the Holstein Association of Canada’s efforts to collect data on linear conformation traits in dairy cattle from the 1970s to the 1980s as a means of improving selection criteria based on type-derived traits [16]. The phenotypic linear traits considered were general appearance, milk form, body capacity, rump, feet and legs, mammary system, fore udder, and rear udder [16,17]. On the other hand, nine descriptive traits were also considered: stature, size, style, brisket, floor, loin strength, dorsal width, and pin placement [12,17]. Heritability estimates for type traits in dairy cattle ranged from 0.06 (style) to 0.33 (size). Phenotypic correlations between type traits and calving interval were essentially zero. The highest beneficial negative genetic correlations with calving interval were for breast depth (−0.42), rear udder (−0.37), and capacity (−0.34), and the highest antagonistic positive correlations were for milk yield (0.43) and milk quality traits (0.38) [17]. Regarding the selection of dairy cows for body size, they usually showed divergence associated with body weight, body dimensions, and calf birth weight but did not differ for milk yield. However, udder conformation in relation to body size differed between large and small cows [16].

In the late 1900s and early 2000s, genomic evaluation methods were developed, allowing for more accurate and efficient evaluations of udder traits. Today, a combination of visual inspection, manual palpation, milk production testing, and genomic and phenotypic evaluation methods are becoming widespread to evaluate udder traits in dairy cattle. Thus, current breeding programs based on the traditional quantitative approach have achieved appreciable genetic gains for milk production. They have implemented selection for other traits, including milk composition, udder morphology, somatic cell count, and mastitis resistance [1,18]. The implementation of novel selection strategies utilizing molecular-based data to enhance both conventional and high-value traits holds significant promise for advancing functional improvements in dairy breeds [1]. Recently, genome-wide association (GWAs)-based studies have demonstrated that udder conformation traits significantly affect animal health, milk production, and the profitability of Holstein cattle [19]. The release of complete genome sequences enabled the use of high-performance genetic markers improving udder trait predictions using genotyping platforms (DNA-derived arrays or SNP chips) [3]. In addition, DNA microarrays (MGEP) began to be widely used in functional genomics or transcriptomics [20]. In the coming years, genomics based on SNP chips or microarrays will possibly be replaced by next-generation sequencing (NGS) technologies [13,21,22]. Finally, the use of ‘omic’ technologies such as epigenomics, transcriptomics, proteomics, metabolomics, metagenomics, and meta-transcriptomics will open new horizons to accurately assess novel udder-derived traits for dairy cattle selection [23]. Overall, the integration of new technologies such as genomics, epigenomics, transcriptomics, proteomics, metabolomics, metagenomics, and meta-transcriptomics is crucial to accurately assess novel udder-derived traits for dairy cattle selection and, thus, improve the prediction of mammary gland characteristics related to udder health, longevity, welfare, and milk production.

## 3. Evaluation Criteria Based on Udder Phenotypic Traits

**Figure 1:**
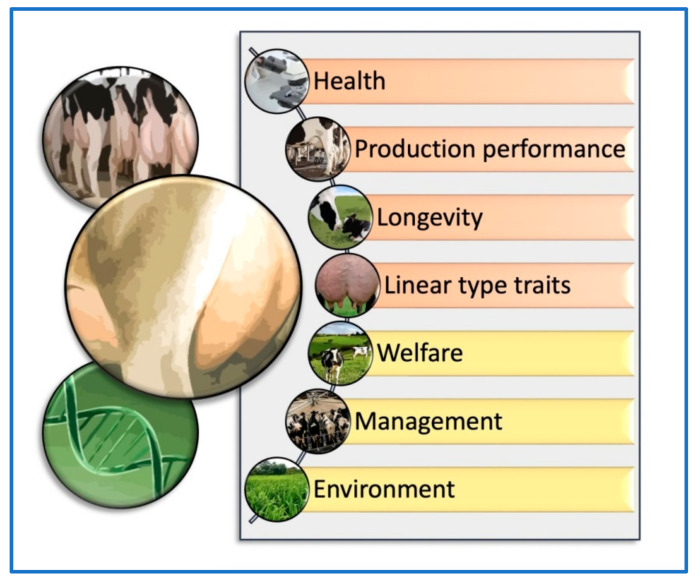
Brief scheme of the main elements that should be considered together with the phenotypic and genomic udder evaluation methods for dairy cattle selection. Orange: intrinsic components such as health, production, longevity, and linear (conformation) type traits. Yellow: extrinsic components such as welfare, management, and environment.

Phenotypic evaluation has long been a key component of dairy cattle breeding, allowing farmers and breeders to evaluate a cow’s physical traits and characteristics to make informed breeding decisions. While more recent advancements in genomic evaluation have provided new tools for evaluating an animal’s genetic potential, phenotypic evaluation remains a critical aspect of the breeding process, particularly when assessing traits that cannot be measured through DNA analysis. However, several intrinsic and extrinsic factors should be taken into account together with the phenotypic and genomic udder evaluation methods for dairy cattle selection (Figure 1). Understanding the criteria used to assess udder phenotypic traits is an essential aspect of dairy cattle breeding, as these traits are key indicators of a cow’s milk production potential and overall udder health. The selection criteria for productive animals based on udder conformation include several traits associated with the general linear conformation of females, such as stature, strength, milk production, teat diameter, hind legs (lateral view), rump angle, rump width, anterior udder attachment, posterior udder height, posterior udder arch, udder depth, suspensory ligament, and teat placement [12]. The selection criteria based on udder conformation traits could differ depending on the livestock species, breed, or crossbreed evaluated. In one study, Montbéliarde (MO) × Holstein (HO) and Viking Red (VR) × HO crossbred cows were compared with first-calving pure HO cows to evaluate the conformation for production and reproductive traits [24]. It was observed that crossbred cows had less udder-to-hock clearance than HO cows. In addition, crossbred cows had more distance between the front and rear teats and longer teats than HO cows [24,25]. However, the frequency of first lactation cows that were discarded because of udder conformation issues was uniformly low across all groups (<1%) [24]. Although this review mainly focuses on dairy cattle, it is worth noting that other ruminant species, such as goats, also have their own linear evaluation systems for assessing individual traits based on their observed range. The American Dairy Goat Association’s linear evaluation system analyzes individual traits in an example of other ruminant species such as caprine by assessing each trait based on its observed range in the animal being evaluated. This is carried out by starting from one biological end of the trait (such as head structure) and evaluating it until reaching the other biological end (such as leg structure). A score or grade ranging from 1 to 50 points is assigned to each trait, and a final type score between 50 and 99 points is calculated based on the importance given to each of the structural categories, which are general appearance (35%), dairy character (20%), body capacity (10%), and mammary system (35%) [12]. In several countries, particularly the United States, genetic evaluations are performed annually for the final type score and additional linear type traits, as in dairy goats [25].

Among the selection criteria taken into account in cattle, the direct measuring and correlated responses to a single trait for milk production could be a convenient method in dairy production systems [26]. For example, several producers use a selection line consisting of artificial insemination (AI) bulls selected for their high estimated transmission capacity for milk production associated with udder conformation [27]. On the other hand, several studies currently target the genetic evaluation of novel traits based on methods to predict selection accuracy. These methods are based on reference populations of cows using indicator traits that could increase the accuracy of genomic selection for important traits such as udder health [3]. Moreover, in other ruminant species, linear or non-linear genetic relationships between functional productive life associated with anterior udder attachment, posterior udder height, posterior udder arch, udder depth, suspensory ligament, and teat location have been described in U.S. dairy goats [12].

Furthermore, heritabilities and correlations between traits were estimated through linear mixed models with additive pedigree genetic relationships and ASREML software (version 2023) [12]. Therefore, udder conformation traits can be used as a selection criterion to increase longevity in dairy goats and can be extrapolated to other species, such as cattle. This consideration of non-linear relationships can help design more efficient breeding programs using conformation traits [12,28]. However, udder conformation in cows with large funnel teats, pendulous udders after calving, and blind quarters presented a higher risk of subclinical mastitis or intramammary infections [29].

Generally, breeders and technicians have considered that there are several traits of importance to consider, such as milk value and udder conformation [30]. Significant interactions indicated that dairy aptitude is the most crucial trait [30]. Subjective visual assessment of dairy cattle by appraisers is carried out to determine various distinguishing characteristics, including linear type traits related to udder conformation [31]. Thus, genetic links between routine observations carried out by appraisers during the evaluation process can be used to validate the classifications from individual appraisers [31,32]. In general, udder central ligament, teat length, udder (in general), and teat placement receive different scores that relate to milk production ability in several cattle breeds [31,33,34].

## 4. Evaluation Criteria Based on Udder Genomic Traits

As genomic evaluation methods have become more prevalent in dairy cattle breeding, understanding the criteria used to evaluate udder genomic traits has become increasingly crucial for breeders seeking to make informed decisions about animal selection. The progress of genetic and genomic evaluation in dairy cattle is based on discovering new traits of interest for predicting selection accuracy. Thus, the genetic and genomic assessment aims to improve how using indicator traits could increase the accuracy of genomic selection [3]. To develop effective selection programs for new traits, it is necessary to create large databases based on highly reliable estimated genetic values (EGVs) [2,35,36]. For evaluating the most costly traits, it is possible to perform extensive phenotyping in combination with genotyping of female dairy cattle to record and characterize their functionality [2]. These novel traits include udder health, hoof health, feed efficiency, methane emissions, and others [3]. There are various deterministic methods to estimate the reliability of genomic evaluations. These depend on factors such as the number of cows used as a reference, the trait’s heritability, and other population characteristics. This is conducted by analyzing the impact of individual genetic markers called single nucleotide polymorphisms (SNPs) [3]. 

When analyzing genetic data, a positive correlation was found between Somatic Cell Score (SCS) and milk quality and composition traits, highlighting the interconnected nature of these characteristics [11,20,23]. The genetic traits for milk composition were negatively correlated with the udder conformation (−0.40) and with rear muscling and udder volume (−0.28) [11]. These results were obtained by using the current selection index, which mainly focuses on dairy strength and could negatively influence all other traits [11]. Moreover, genetic correlations between udder traits are favorable, indicating that selection for a given trait will positively affect the evolution of overall udder conformation. In particular, the degree of udder suspension was highly correlated with udder depth (0.82) [26]. Genetic correlations with milk production were low and negative, except for udder depth (−0.48) [26]. Accordingly, more appropriate selection indices based on genetically derived traits should also be considered, together with functional traits [37]. Recently, a more detailed report on genomic methods and general conformation traits applied for dairy cattle selection was published [13]. However, this report did not thoroughly consider the specific udder conformation and functional traits and their relationship with udder-related genomic traits. Udder conformation is not only crucial for displaying visual characteristics but also for high milk production and low incidence of mastitis. A recent study measured different udder type traits in Sahiwal (*Bos indicus*) and Karan Fries (*Bos taurus × Bos indicus*) and their association with SNPs in vitamin D receptor and protein tyrosine phosphatase, receptor type (*R*) genes. The *GG* genotype of SNP *rs454303072* was associated with a wider rear udder, larger udder circumference, and longer distance between the front and rear teats and left and right teats in Karan Fries cattle. On the other hand, in Sahiwal cattle, the *AA* genotype of this SNP was found to be associated with a higher and wider rear udder, larger udder circumference, and wider udder. In addition, the *AA* genotype of the SNP *rs382671389* was associated with longer front teats in Karan Fries cattle. The *TT* and *CC* genotypes of SNP *rs435289107* were associated with udder-type traits in Karan Fries and Sahiwal cattle, respectively. These results suggest that *BTA5* harbors genomic regions associated with udder traits in *Bos indicus* and *Bos indicus x Bos taurus* cattle [38].

All data based on genome sequences from previous generations provides comprehensive information on the polymorphic loci that characterize a given breed and differentiate it from others. Association analysis with imputed sequences, mainly when applied to multiple traits simultaneously, is a powerful approach to detecting candidate causal variants underlying complex phenotypes. Twelve quantitative trait loci (QTL) affecting different morphological characteristics of the mammary gland were detected in the German Fleckvieh cattle population. Most of the QTLs were located in non-coding regions of the genome but in close proximity to candidate genes that could be involved in mammary gland morphology (*SP5, GC, NPFFR2, CRIM1, RXFP2, TBX5, RBM19*, and *ADAM12*) [39]. In Holstein cattle, genetic analyses of udder conformation traits have been developed [19]. A genome-wide association has been described with five udder conformation traits, including anterior udder attachment, central suspensory ligament, posterior udder attachment height, posterior udder attachment width, and udder depth [19]. Heritability and standard errors for these five udder traits ranged from 0.04 ± 0.00 to 0.49 ± 0.03. In this study, phenotypic data were measured on 1000 Holstein cows, and the GeneSeek Genomic Profiler (GGP) Bovine 100 K SNP chip was used to analyze genotypic data [19]. Numerous candidate genes were identified within 200 kb of significant SNPs. Among all significant SNPs associated with udder conformation traits, most of them were located within the following genes: Microsomal Glutathione S-Transferase 1 (*MGST1),* Microsomal Glutathione S-Transferase 2 (*MGST2),* Microtubule-associated scaffold protein 1 (*MTUS1), LOC101903734*, *LOC112447118*, Structural maintenance of chromosomes protein 5 and 6 (*SMC5-SMC6)*, Parkin RBR E3 ubiquitin-protein ligase (*PRKN),* Syntaxin-Binding Protein 6 (*STXBP6),* glutamate ionotropic receptor delta-type subunit 2 (*GRID2),* E2F transcription factor 8 (*E2F8),* Cadherin 11 (*CDH11),* Forkhead Box P1 (*FOXP1),* Localisation factor complex 1 (*SLF1),* transmembrane protein 117 (*TMEM117),* SET binding factor 2 (*SBF2),* CGPVitamin D binding protein (*GC),* galectin 2 (*LGALS2*), adhesion G protein-coupled receptor B3 (*ADGRB3)*, and Glutamate-Cysteine Ligase Catalytic Subunit *(GCLC)* as well as *KEGG* pathway genes. While one of the SNPs on *Chr6* was close (50 kb) to the ubiquitin-conjugating enzyme E2 K gene (*UBE2K*), three SNPs on *Chr21*, *Chr29*, and *Chr18* were located close (100 kb) to *STXBP6*, *E2F8*, and *CDH11*, respectively [19]. These results could provide valuable biological information for the genetic structure of udder conformation traits in Holstein dairy cattle [19]. 

Several studies have identified quantitative trait loci (QTL) to identify and understand genetic variants associated with economically relevant phenotypes. However, to identify QTL and genomic regions associated with phenotypes in Holstein cattle, the GWAS method has become the most widely used, proving to be an effective tool for identifying genetic variants associated with economically important traits [40]. 

## 5. Evaluation Criteria Based on Genomic and Phenotypic Traits: Udder Health, Production, and Longevity

### 5.1. Genomic, Genotypic, and Phenotypic Udder Traits: Impact on Mammary Gland Health

The development of national health registration programs began several decades ago. Accordingly, the first reports of genetic results based on health traits in the United States started in 2004 [41]. A few years later, in 2007, Canada reported large amounts of data on mastitis incidence in cows [42,43]. Available data based on the mastitis incidence can be analyzed by multi-trait models using SCS data and other derived traits indicative of SCS in early lactation, partial SCS, test day SCS, anterior udder attachment, udder depth, and BCS to perform traditional and genomic evaluations for mastitis resistance [35,43,44]. Udder health traits associated with bacteriological testing of quarters maximize the information on infection; however, these tests are not practical on a population scale [45]. In addition, somatic cell counts (SCCs) are highly variable, difficult to interpret, and not sensitive indicators of subclinical infections [45].

Nevertheless, several milk enzymes are possible indicators of tissue damage at the mammary gland level [45]. The heritabilities are around 0.2 for SCCs and 0.1 for other traits, reflecting genetic variations in the immunological defenses and their effect on the teat, phagocytosis, and immune response [45]. There is a positive relationship between somatic cell count (SCC) and milk yield at the genetic level but a strong negative relationship between them at the phenotypic level [45]. Genetic correlation refers to the degree to which the same genes affect two different traits, in this case, SCC and milk yield. A positive genetic correlation between SCC and milk yield means that the genes contributing to higher milk yield also contribute to higher SCC.

In several countries, the genomic values for mastitis resistance have been adopted as part of national selection indices [29,36,42,46,47]. A recent study based on milk production traits aimed to identify genes associated with mammary conformation and health phenotypes in Holstein, Montbéliarde, and Normande breeds [48]. Cows were genotyped with medium (50 k) or low (7 to 10 k) density chips and imputed to 50 k, resulting in 1013 genes associated with udder morphology, mastitis, and production phenotypes (e.g., *ESR1*, *FGF2*, *FGFR2*, *FGFR2*, *GLI2*, *IQGAP3*, *PGR*, *PRLR*, *RREB1*, *BTRC*, and *TGFBR2*, among others) [48]. Another study identified potential candidate genes for novel udder health traits targeting specific mastitis pathogens and Holstein Friesian cows’ differential somatic cell fractions. Thirty-five significant SNPs were detected on 14 different chromosomes for cell fractions and pathogens. In this case, depending on environmental stressors, the HEMK1 gene plays a role in developing the immune system.

Furthermore, it was observed that the *CHL1* gene is up-regulated with stress levels and influences the mechanisms of the immune system [49]. Therefore, the activity interactions between the genes may fluctuate depending on environmental stressors. On the other hand, other authors report that *CFAP69*, *STEAP2*, and *ITGB3BP* genes located on chromosome 4 are related to the mechanisms of the mammary gland immune system [50,51]. The objective measures of mammary gland conformation and linear type scores are used to predict mastitis in experimental linear classification programs [1]. However, the relationships between conformation and mastitis are often inconsistent due to moderate or low correlations between mastitis indicators [46]. Selection to reduce the occurrence of cows with deep udders, especially low rear udders, open teats, teats that are set back, and also short and wide teats, can improve efforts to reduce the incidence of mastitis, along with better health control, therapeutic managing, and proper milking procedures [46]. A study in the U.S. reported genetic analyses performed for health traits, including reproductive problems, metabolic diseases, lameness, and the incidence of mastitis [36]. This study concluded that genetic selection for health traits using recorded phenotypic data and including genomic data would substantially improve selection accuracy [36]. In addition to the mentioned traits, others, such as milk electrical conductivity, are available to help predict mastitis incidence [52]. Conformation traits in Viking Red (VR), Montbéliarde (MO), and Holstein (HO) crossbred cows in commercial dairy herds were associated with significantly lower health indices during the first (−23%), second (−29%), and third (−21%) lactation comparing the VR and MO group with the HO group [14]. Crossbred cows had significantly less udder to hock spacing but significantly wider rear teat width and longer teat length compared to the HO group [14]. It is important to note that in the least-developed countries, the national phenotypic, genetic, and genomic evaluations for udder and other health traits are not currently being planned. It is, therefore, essential to establish national udder health registration programs to facilitate future selection protocols [35,36,53]. 

There are factors associated with the distribution of clinical bovine mastitis between the hind and forequarters, in addition to risk factors related to certain aspects of lactation, udder conformation, and management practices [54,55,56]. Thus, programs to select sires whose progeny have the lowest SCCs should be carefully planned, taking into account the interpretation of SCCs as an immunological defense mechanism [11,27,57,58]. High SCCs decrease the likelihood of subsequent infections; however, there is a heritable variation in SCCs in dairy cattle. Therefore, using SCCs as a defense marker should be studied in greater depth [45]. Clinical mastitis in the hindquarters was found to be more common among primiparous cows, with a prevalence rate of 61.9% in affected cases [54]. Moreover, udder conformation does not seem to play an essential role in mastitis incidence and distribution [37,46,59]. In another study, the genetic traits of milking speed (subjectively scored) and SCS estimation using Restricted Maximum Likelihood (REML) were evaluated [58]. The results showed that heritabilities were 0.15 for milking speed and 0.14 and 0.16 for mean SCS in the first and second lactation, respectively [58]. Genetic correlations between milking speed and SCS were 0.41 and 0.25 for the first and second lactation, respectively, indicating that faster milking was associated with increased SCS [58]. The genetic correlations were higher between udder depth and SCS (−0.26) and between rear udder width and milking speed (−0.24). Therefore, an udder health index applied to sire selection was developed for an aggregated genotype, including subclinical mastitis in 1 and ≥2 lactations and clinical mastitis in 1 and ≥2 lactations concerning milking speed [58]. Finally, the genetic and phenotypic correlations between type traits, heritability indices, and arithmetic mean SCCs during lactation were determined using a minimum norm quadratic unbiased estimator (MINQUE) and two multiple trait REML methods [57]. The heritabilities obtained for all traits were low. Regarding SCCs, heritabilities ranged from 0.09 to 0.11, and for type traits, from 0.08 to 0.14. The genetic correlations between SCCs and type traits ranged from −0.22 to 0.30; however, the phenotypic correlations were very low. In general, the correlations indicate an association between SCCs and udder conformation traits; that is to say, a desirable type score would be associated with a low SCC [57]. In summary, the traits included in the health index were milking speed, udder conformation, and SCS in the first and subsequent lactations [58]. The accuracy of the health index was found to be 0.776. This was a 15% increase compared to an index that was based on SCS count alone [58].

Prior studies anticipated that variations in udder infection rates could be linked to udder conformation and milkability [60]. The number of infected quarters was 1.53 for the low infection rate group and 2.53 for the high infection rate group [60]. Poor udder and teat conformation were associated with high levels of intramammary infection, as indicated by increased SCCs [9,55,56,61]. In addition, both udder physical traits and SCCs were related to the productive life in sheep and cattle [61]. Udder conformation and teat end lesions have been associated with risk factors such as elevated SCCs and intramammary infections in dairy cows [31,46,55]. Cows with large udder hindquarters had significantly fewer *Staphylococcus aureus*- and *Streptococcus uberis*-derived infections [54,55]. There was a similar, although less significant, negative association with *Escherichia coli*-derived infection [55,62]. *Streptococcus agalactiae-* and *Streptococcus dysgalactiae*-derived infections were more frequent in cows with large pendulous or small udder conformations [55]. Moreover, anatomical teat characteristics (length, cylinder diameter, and tip) in dairy cows were associated with intramammary infections caused by teat skin bacterial populations [56,61,63,64]. Because of this, linear conformation data have been evaluated for udder type in dairy cattle, including teat position (front or rear), which is associated with the occurrence of clinical mastitis [56]. In addition, *GAS6* negatively regulates the *Staphylococcus aureus*-induced inflammatory response in the mouse mammary gland [65]. Finally, the *NCAM1* gene, also known as *CD56*, is a member of the immunoglobulin superfamily and is the phenotypic marker of natural killer cells [66].

Other udder-related pathologies, such as udder cleft dermatitis (UCD), could be considered a trait affecting udder health and conformation [47]. Dermatitis is an inflammatory skin condition associated with mastitis and digital dermatitis that affects the anterior parts of the udder in dairy cows [47]. The factors with the most significant effects on such lesions were days in milk, milk production, and early calving [47]. The etiology of UCD is probably multifactorial, involving udder conformation traits, other cow-related aspects, and herd-related risk factors [47]. The high prevalence of severe UCD lesions in dairy cows from Nordic countries such as Sweden emphasizes the need for preventive genomic and phenotypic evaluations together with efficient treatments to avoid the occurrence of UCD in future dairy cattle [47]. 

Recently, eight different machine learning methods were compared to predict the udder health status of cows based on somatic cell counts. The study found that all methods had prediction accuracies above 75%. Neural Network, Random Forest, and linear methods best predicted udder health classes based on recorded milk traits. The findings suggest that machine learning algorithms can be a promising tool to improve farmers’ decision-making in identifying cows with high somatic cell counts, which can improve surveillance methods and potentially reduce the economic and health impact of bovine mastitis in dairy farms [67,68]. The use of machine learning and data mining techniques in genomic and phenotypic udder evaluation for dairy cattle selection has become an essential tool in improving udder health. Several research groups have been investigating the implementation of these methods [67,69,70,71]. Furthermore, integrating both genomic and phenotypic information has been shown to significantly improve the accuracy of predictions regarding udder health in dairy cattle.

### 5.2. Genomic, Genotypic, and Phenotypic Udder Traits: Effects on Milk Production and Quality

Traditionally, dairy cattle have been selected for their ability to produce milk in quantity and quality [20]. The traditional approach to dairy cattle selection has changed, and secondary traits are now included in the selection indices, with less emphasis on milk production and more interest in milk quality and other non-productive traits [20,53,63]. Greater emphasis on non-productive traits is reflected in the industry’s desire to breed less productive but more functional dairy cattle [20]. According to some studies, there is a low but statistically significant relationship between conformation traits and the lifetime production efficiency of cows (Guilford scale), being slightly higher for milk production than longevity [72]. Type and conformation traits seem to be more suitable for predicting the lifetime production efficiency of cows compared to other more detailed traits [17,25,72]. For example, lifetime performance was most strongly related to overall linear score and udder linear score (r = 0.22), followed by type and conformation scores, feet and hooves (r = 0.13), and, finally, other detailed traits such as udder width and dairy character (r = 0.14) [72]. Therefore, type and conformation traits seem to be more suitable than genomic traits for predicting the lifetime production efficiency of cows [11,72]. 

The phenotypic and genomic traits related to milk yield, protein, and fat are included in the performance tests in young bulls [8,11]. Several linear-type traits have been added to the factor analysis. Heritability (h2) estimates for milk yield and milk quality-derived traits ranged from 0.125 to 0.219 [11,45]. Moreover, genetic estimates for milk yield and milk quality traits were negatively correlated with traits explaining udder conformation (−0.40) and rear muscularity [11]. The latter genetic traits also showed a negative correlation with udder volume (−0.28). Additionally, head typicality and rear leg traits were not correlated with milk yield and milk quality but were negatively correlated with meat-related traits such as rear muscularity (−0.32) [11]. The consequence of these results is that the use of the current selection index, which is mainly focused on milk production traits, may lead to a deterioration of all other traits. Therefore, more appropriate selection indices should consider an association between genetic and functional traits [11,23,37,57]. A previous study revealed that 51 significant SNPs overlapped with 51 protein-coding genes together with five QTL for SCC. On chromosome *BTA18*, three QTL for SCC overlapped with SNP *rs41622425*, which is located within the intron of a gene encoding a protein called Pleckstrin Homology-Like Domain Family B Member 3 (*PHLDB3*). In addition, on the same chromosome, *rs110437995* overlapped with QTL for SCC. They also detected three overlapping genes, GAS6, PKD2, and NCAM1, previously associated with inflammation in humans and mice [73].

The selection and correlation of different traits for milk production based on the selection of AI bulls preferred for high transmission capacity is now possible [3,27,57]. The estimates regarding sire selection criteria may vary; however, the selection for milk production traits effectively increases milk production [8]. Nonetheless, in a research project evaluating the direct and correlated effects of single-trait selection on milk yield all selective breeding groups increased productivity, but also undesirable responses correlated with selection for milk production were detected [27]. Sometimes, bull sires from different genetic lines were selected by progeny testing based on the first lactation productivity and milk quality by associating traits for fat-corrected milk production, percentage of daughters discarded at the first lactation, and daughters’ udder conformation. However, one study showed no differences regarding milk yield among lines [57]. Moreover, negative correlations existed for milk production traits and quality traits without affecting udder conformation traits for the selection lines considered. Additionally, cows selected for the higher production traits tend to have higher health costs, specifically for mammary treatment [27,74]. Finally, other factors, such as chronic stress, can affect cow welfare, and acute stress during milking can decrease milk production [6,37]. Therefore, it is important to quantify whether long-term stress persistence would impact milk production depending on the genetic line studied [6]. Another study compared the reproductive performance, milk yield and composition, udder health, and conformation traits of Holstein, F1, and R1 crossbred Holstein × Simmental cows [75]. Milk yield and fat-protein yield did not differ between groups [75]. Furthermore, udder conformation traits lacked high scores in crossbred cows. Finally, Holstein cows had significantly shallower udders and higher udder spacing than the other groups [75]. 

It has been observed by RNA sequencing that *DDIT3*, *RPL23A*, *SESN2,* and *NR4A1* genes are significantly and differentially expressed between mammary glands of lactating Holstein cows with extremely high or low protein and fat percentages. Therefore, these four genes could affect milk production and composition traits [76]. In addition, the genes identified in another study, such as *PGM1* and *ARL4A*, were related to milk production traits, lactose synthesis, glucose metabolism [77], and milk production or composition [78]. Finally, the *ROR1* gene located on chromosome 3 has been considered a causal determinant gene for somatic cell count [79]. These studies shed light on the importance of genetic factors in determining milk production and composition traits and highlight the need for further research in this area to improve the efficiency and profitability of the dairy industry.

Linear evaluation plays a vital role in estimating the milk production of dairy cows; however, these evaluations, sometimes taken through subjective methods, can show variations [5,31]. On the other hand, objective methods for estimating milk productivity provide more accurate and reliable data but require more sophisticated technologies [5]. In one study, sire-derived type traits were analyzed in cows with low, medium, or high production performance during the first lactation [80]. The factors examined in the model included herd-year-season, age at calving, the month of calving, recording status interaction, change in herd size, and season. In addition, fat and protein production and type-based sire linear regressions were analyzed within each group of cows with similar production traits [80]. The impact of leg traits on the number of lactations was almost the same as for udder traits. In addition, interactions between traits were significant. Conformation-type traits were of little importance for herd life in low-producing cows. However, few differences were observed in the relationships between herd life and type in medium- versus high-producing cows, indicating that there is no need to increase emphasis on type in response to current trends for higher production [80]. These studies reinforce the idea that a comprehensive and detailed analysis is necessary to estimate milk production in dairy cows accurately. Furthermore, a primary goal of modern cattle breeding is to attain an equilibrium between various phenotypic traits and overall functions linked to mammary gland conformation, which can effectively manifest desirable, productive traits in diverse environments [1,3,11,59,80].

### 5.3. Genomic, Genotypic, and Phenotypic Udder Traits: Influence on True and Functional Longevity

Longevity-related traits have garnered growing interest among various milk-producing species, including sheep, cattle, goats, and other animals, as efforts to enhance the longevity of these animals continue to gain importance. Therefore, understanding the impact of genomic, genotypic, and phenotypic udder traits is crucial in determining dairy cows’ true and functional longevity. Functional longevity is defined as the number of days between first calving and culling, that is to say, the length of cows´ productive life [81]. Functional longevity is an economically important trait to increase the profitability of dairy management [82]. In different bovine dairy herds, the reasons for culling cows can be voluntary (mainly because of low productivity) or involuntary (mainly because of health and low fertility) [81,83]. The longevity-derived traits include (i) true longevity (all reasons for culling, including productivity) and (ii) functional longevity (all reasons for culling except productivity) [82]. Conformation traits and functional longevity have been related by survival analysis (Cox proportional hazards models) in first-lactation Holstein cows [81]. The dairy character had the strongest correlation between a composite trait and functional longevity, followed by the udder final score [81,84]. Among the descriptive traits, the highest correlations were observed for traits related to longevity vs. udder attachment and longevity vs. udder depth [81,83]. Cows with deep udders had significantly lower functional survival than cows with shallow udders [75]. The highest positive effect on longevity was exerted by udder score and legs and hooves (r = 0.11), udder placement (r = 0.14), and anterior udder attachment (r = 0.10) [72]. In addition, central ligament weakness was associated with significantly reduced cow longevity [81].

A more accurate methodology for improving animal longevity is necessary to decrease the involuntary culling rates rather than extending traits that influence the herd life [82]. Therefore, the proportional hazard model is helpful for assessing genetic fitness for the traits influencing herd life. However, the differences between estimates made with the proportional hazards models and those made with linear animal models for one or more traits are unclear. Productive traits, udder traits, and leg and hoof traits are genetically correlated with longevity; consequently, these traits are used to assess longevity indirectly [12,16]. The reliability of genetic fitness-related estimates for longevity is increased by combining direct and indirect estimates [82]. Therefore, these genetic correlations should be reviewed periodically in different dairy cattle production systems as they vary according to the year of birth [82]. In light of this, QTLs affecting economically relevant traits were studied for eight US Holstein cattle genetic lines [85]. A marker on chromosome 14 associated with differences in fat yield, fat percentage, and milk yield was observed in two genetic lines. Other markers located on chromosomes 16 and 20 were related to differences in udder depth and anterior udder attachment, respectively. A marker on chromosome 27 was associated with a difference in milkability index. These additional markers complete the quantitative trait locus mapping to identify QTLs affecting economically important traits in a selected commercial Holstein population [85]. Another study in dairy cattle demonstrated the association of SNPs located within selected genes with traits associated with longevity [86]. In summary, the polymorphism with the most significant effect on functional longevity is leptin (*LEP-R25C*) in dairy cattle [86]. In addition, four potential candidate genes, *ETV1*, *ONECUT1*, *MACROD2*, and *SIRT1*, have been identified in Holstein cows that directly (through disease resistance mechanisms) or indirectly (through milk productivity) influence longevity [87]. Finally, the three bovine selectin-related genes identified as *SELP*, *SELL*, and *SELE* have been described as relevant genes for udder traits, with *SELP* being the most polymorphic of these genes. It is suggested that these polymorphisms in *SELP* and *SELE* are associated with fertility, milk production traits, and longevity in Holstein Friesian dairy cows [88].

The relationship between different conformation traits and functional longevity is essential in dairy cows and has been evaluated for years using survival analyses [80,81]. As mentioned, the highest correlations among descriptive traits were observed for longevity-udder attachment and longevity-udder depth [26,35,43,46,59]. However, functional longevity decreased with decreasing body condition in dairy cows [81]. Other relevant factors affecting longevity could be related to chronic stress in dairy cattle. For example, environmental factors can affect genomic and phenotypic traits in the dairy cow [6]. The transition of a dairy herd to an automatic milking system from a conventional parlor system can also be stressful for the cow, as many changes occur during this process [6,37]. The robotic milking methods applied to dairy cows will be essential tools that will probably be introduced, as a general rule, in all dairy farms [89]. However, there is a substantial possibility that the robotic methods may not be able to be adapted adequately to the spatial location of the teats [89]. It has been found that robotic attachment is more successful in cows whose udder conformation is more suitable. Moreover, the cows showed some signs of discomfort after the omission of milking when robotic milking systems were used [6,89]. In 60% of cases, there is milk loss after robotic milking, which indicates ineffectiveness and additional risk for mastitis, affecting longevity [89]. Therefore, special attention should be paid to the conformational udder-related traits in cows destined for robotic milking that will be useful for dairy cows with high performance, good udder conformation, health, and longevity [3,9,81]. The use of mechanical milking has led to the selection of animals based on udder morphology (linear scoring) [12,25]. In order to improve longevity, teat placement, degree of udder suspension, udder depth, and the degree of separation of the two halves are evaluated using nine-point linear scales [26,90]. It has also been observed that the linear combination of individual linear traits is crucial for longevity and is highly correlated with teat placement, degree of udder suspension, and udder depth, with repeatability across lactation showing a value of 0.76 [26]. 

Dairy cows have a short productive life, so longevity-derived traits are essential from an economic perspective [53]. Combining a long lifespan with high functionality could help improve dairy cow productivity [2,91]. Furthermore, genetic values for milk production, fertility, udder health, and leg health are indicators that should be positively associated with the probability of achieving long-lifetime productivity [53,91]. In conclusion, to increase the lifetime of milk production in dairy herds, producers should select heifers with high scores for conformation traits involving udder, feet, and hooves. In addition, they need to consider high genetic values for milk production, fertility, and udder health [53,92].

### 5.4. Relationship between Genomic, Genotypic, and Phenotypic Udder Traits: Health, Production, and Longevity

**Figure 2:**
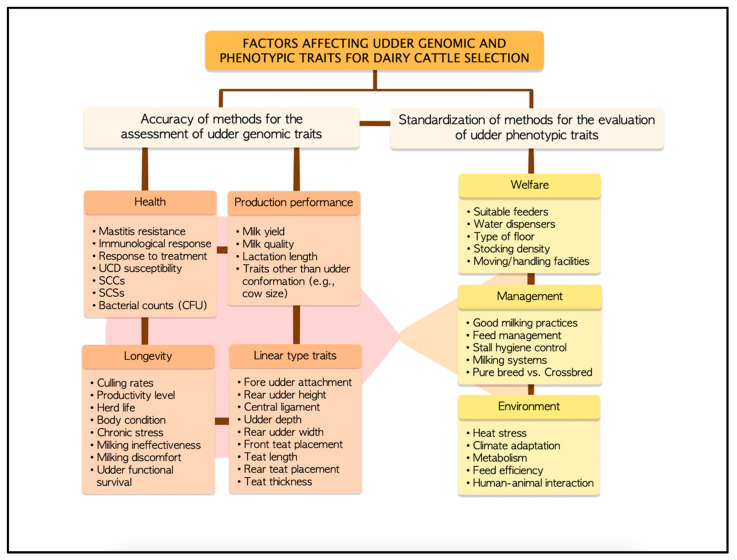
Overview of the factors affecting udder genomic, genotypic, and phenotypic traits for dairy cattle selection. Orange squares on the left: factors affecting udder health, production, and longevity depending on linear (conformation) type traits. Yellow squares on the right: factors affecting udder function depending on welfare, management, and environment-related traits. Both direct and indirect factors are interconnected as they depend on each other and vice versa. Improving dairy herd selection in the medium- and long-term requires the development of cutting-edge prediction methods for udder conformation and function, whether genomic or phenotypic, which underscores their critical importance. Under each factor topic (Health, Production, Longevity, Linear type, Welfare, Management, and Environment), examples of relevant factors are listed. UCD: Udder Cleft Dermatitis; SCCs: Somatic Cell Counts; SCSs: Somatic Cell Scores; CFU: Colony-Forming Units.

The intricate interplay between genomic, genotypic, and phenotypic udder traits is fundamental to understanding the complex relationship between health, production, and longevity in dairy cattle (Figure 2). The dairy cattle sector is significantly impacted by the economic costs associated with the high incidence and prevalence of clinical and subclinical mastitis, including expenses related to treatment, production losses, and reduced animal welfare [59,93]. The large databases generated have allowed for assessing the incidence of this health problem and investigating the genetic background of clinical mastitis and its relationships with other udder-derived traits of interest for the dairy industry [59]. There is persistent controversy about low milk SCCs and susceptibility to mastitis [94]. However, high SCCs in milk may indicate inflammation or infection of the mammary gland [94]. Several studies described that cow selection for mastitis resistance is often based on genetically correlated indicator traits, such as SCCs, udder depth, and attachment [59,94]. Moreover, genetic correlations showed a significant relationship between clinical mastitis, SCCs, and udder depth during early lactation [59]. Therefore, SCCs are also a reference trait for selecting dairy ruminants less prone to mastitis incidence [59]. The selection programs favoring animals with fewer SCCs are still widely debated [59]. However, the divergent selection of animals based on lower SCCs could solve future udder-related health problems [94,95]. It is important to note that the susceptibility to mastitis linked to SCCs is a phenotypic trait that may be related to immunomodulation but not to selection [75]. Genetic correlations between clinical mastitis and other economically important traits indicated that selection would also improve resistance against other diseases, improving fertility and longevity [51,74]. However, milk production remains positively correlated with clinical mastitis, highlighting the importance of including health traits in phenotypic selection programs to achieve genetic progress for all relevant traits [51,74]. Thus, implementing new genomic evaluation models in phenotypic-related studies is expected to enhance the accuracy and efficiency of selecting dairy cattle with improved mastitis resistance [22,34,39]. The genetic variability of mastitis resistance is well established in dairy cattle, with many studies focused on the estimation of heritabilities and genetic correlation between mastitis-related phenotypic traits such as SCCs and clinical cases [36,77,78,79,80,81,82]. 

Other studies involved molecular genome mapping results which provided information on quantitative trait loci (QTL) related to mastitis resistance and provided a better understanding of the genetic relevance of the traits [37]. Many countries have implemented selection programs based on a linear decrease in SCCs for increasing mastitis resistance [37]. Improving the selection accuracy for mastitis resistance includes advances in modeling, an optimal combination of mastitis-related traits, and associated udder predictors [37]. In addition, the definition of the overall breeding objective that includes udder-related conformational and functional traits and the inclusion of molecular-based information is now available from QTL [37,96]. These cutting-edge studies will lead to a better understanding of the genetic background of mastitis resistance and allow for more accurate selection, improving udder health, animal welfare, and profitability in the future modern dairy industry [59,97]. Other potential future studies on mastitis resistance will be based on bacteriology as new indicator traits and tools for dairy cattle selection [59]. The relationship between cows and microorganisms has traditionally been related to intramammary infections [64]. However, recent metagenomic studies indicate that mammary secretions from clinically healthy quarters may harbor genomic markers for detecting several bacterial groups, the majority of which were not associated with mastitis but were defined as “commensal mammary microbiota” [64,98,99]. Whether disruption of udder microbiota diversity (dysbiosis) plays a role in determining susceptibility to mastitis is still unknown, and little is known about the contributions of various biotic and abiotic factors on the composition of udder microbiota diversity [64,100,101]. Therefore, there is a need to study the teat end, teat canal, and mammary secretions-related microbiota as interconnected niches of a highly dynamic microbial ecosystem [64,102]. In addition, it is necessary to analyze host-associated factors, including physiological and anatomical traits, as well as genetic traits that may affect the udder microbiota [64,103]. 

Within the domain of conformation traits, a study was conducted to assess the impact of udder morphological characteristics on milk production in *Bos indicus* cows [104,105]. First, the udder diameter and height, teat length and diameter, and milk production were measured, and finally, the study determined the values of udder morphological characteristics in local zebu cows. The results showed that the udder size was highly and positively correlated with milk production. These findings will be instrumental in genetic improvement programs for zebu cows [106]. In studies related to *Bos taurus*, it has been observed that udder morphology, as well as teat tip length and diameter, play a significant role in the incidence of intramammary infection among Jersey crossbred cows in warm and humid climates [63]. Moreover, pendulous udder, flat and inverted teat tip, and very long and thick teat characteristics were associated with higher susceptibility to intramammary infection in Jersey crossbred cows. Therefore, these traits should be considered relevant when selecting dairy cows [63].

On the other hand, the influence of udder and teat conformation and size on the teat-seeking behavior of newborn calves is a determining factor for calves suckling for the first time [23,30,107]. A shorter distance between the udder and ground (low udders) increased the time spent searching for teats and the time of first suckling, which also significantly affected calf survival [107]. Finally, longevity is an increasingly important trait in dairy and beef cattle cows [61,62]. Increased longevity reduces costs and raises incomes for the producers [63]. The phenotypic relationship between conformation-type traits and longevity in cattle estimates the effect of voluntary culling [61,63]. It has been described that udder conformation had no impact on longevity in beef cattle [63]. However, muscle-derived traits were the most important factors for longevity in beef cows [63]. Therefore, relevant udder-derived traits in dairy cattle differ from those applicable to beef cattle. 

Dairy cattle offer an attractive model for investigating the genes responsible for the substantial diversity observed both within and between mammalian species concerning their milk volume, protein, and fat composition, highlighting the potential significance of genomic traits. Many phenotypes for these traits and the complete genome sequence of the key founders of modern dairy cattle populations are available. Association tests were conducted on Holstein and Jersey cattle with exceptional phenotypes to identify variants within the target regions, while gene expression data were analyzed to pinpoint potential candidate genes such as *BTRC, MGST1, SLC37A1, STAT5A, STAT5B, PAEP, VDR, CSF2RB, MUC1, NCF4*, and *GHDC* associated with milk production [108]. In *Bos taurus*, 141 and five novel genes related to milk production and SCS have been identified, respectively. These novel genes were also found to be functionally related to heat tolerance (e.g., *SLC45A2, IRAG1*, and *LOC101902172*), longevity (e.g., *SYT10* and *LOC101903327*), and fertility [109]. In a previous study, polymorphisms in the gene regulating the osteopontin OPN (*SPP1*) were found to be associated with milk yield traits and somatic cell score (SCS), a trait used to estimate the genetic value of udder health in dairy cattle [110]. Several genomic regions have been found to contain genes, including *ZNF683, DHX9, CUX1, TNNT1*, and *SPRY1,* that may biologically contribute to mastitis development and whose functions range from regulation of cell proliferation to immune system signaling [111]. Genetic investigation of the composite risk trait involved a new locus and candidate genes that have potentially pleiotropic effects related to mastitis [111]. On the other hand, several studies have identified genetic factors related to mastitis resistance or susceptibility in dairy cows. Classical traits related to mastitis include somatic cell counts, electrical conductivity, and clinical cases of the disease. However, new traits such as acute phase proteins, immunological assays, and milk flow patterns are considered by discovering new biological pathways, e.g., the role of the mammary epithelium and the nervous system and their relationship to disease development [112]. Furthermore, the usefulness of these traits for genetic improvement has been determined [113]. An additional study detected genomic regions that hold potential candidate genes linked to mastitis resistance and susceptibility, immune system and inflammation, milk properties, udder morphology, and various cancer types situated on a segment of chromosome 8 (SNP rs41609496). Among the genes within this segment are AKNA, MIR455, ORM1, and GNG10, which play crucial roles in mammary gland immunity and are relevant to milk production [112]. 

## 6. Perspectives and Future Challenges for Genomic, Genotypic, and Phenotypic Udder Evaluation

**Figure 3:**
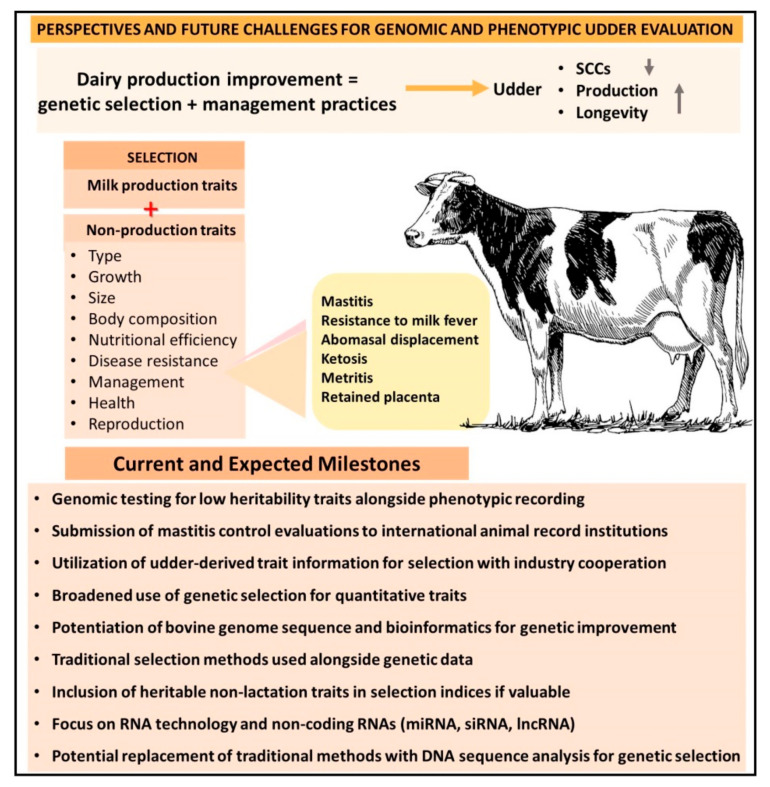
Scheme of the perspectives and future challenges for genomic, genotypic, and phenotypic udder evaluation. Current and expected milestones should be considered for the improvement of dairy cattle selection. miRNA: micro-RNA; siRNA: small interfering RNA; long non-coding RNA.

Several secondary traits have been included in the selection indices, decreasing the emphasis on the milk yield-derived trait [114]. The dairy cattle industry has recognized the importance of including non-production traits in the selection process of functional dairy cattle. These traits include type, growth, size, body composition, nutritional efficiency, disease resistance, udder health, reproduction, and management (Figure 3). Most of these traits can be found within selection indices worldwide, although the relative importance of each trait is variable [115]. For example, the non-lactation traits mentioned above are quantitative, where the whole animal’s phenotype represents the sum of minor traits [20].

The development and implementation of genetic evaluations for health traits have led to an accelerated pace for incorporating new functional traits for dairy cattle selection. Since 2018, the Council on Dairy Cattle Breeding (CDCB, Bowie, MD, USA) has made routine official genomic evaluations available to producers for six health-related traits in Holstein cattle [23]. These traits include resistance to milk fever, abomasal displacement, ketosis, clinical mastitis, metritis, and retained placenta. The genetic merit indices include these health-related traits [23]. Improving cow health is mainly carried out through changes in management practices and genetic selection based on SCSs, productive life, and longevity [23,116]. Genomic testing allows an accelerated improvement of traits with low heritability, such as health traits; however, phenotypic traits remain essential for successful genomic evaluations associated with data where appropriate quality control standards have been applied [23,117]. Mastitis control evaluations are submitted to Interbull together with SCSs for international validation and udder health evaluation [23]. In addition, the evaluation includes data from different breeds regarding health traits, using multiple trait models and additional functional evaluations of new traits such as calf health and feed efficiency [23,118].

In the present era, a wealth of information can facilitate informed decisions for the selection of udder-derived traits with greater accuracy than ever before; however, the development of future decisions will require new and ongoing cooperation among livestock industry stakeholders [59]. Therefore, it is crucial to provide accurate and unbiased information related to udder-derived and other production-related traits [23,119]. Functional traits based on direct information about cow health have also become more critical due to increasing concerns about animal welfare and consumer demands for healthy and natural products [2]. Genetic selection can be applied to quantitative traits, but it is challenging to link successful genetic selection to physiological traits [20]. The bovine genome sequence will have an essential role that should not be underestimated in the future of dairy cattle [20,120]. Completing the bovine genome sequence was the first step towards modernizing our approach to dairy cattle genetics [20,121].

Nevertheless, finding functional genes is complex, and scanning billions of DNA base pairs is expensive and time-consuming [20,122]. Moreover, the function of most genes is unknown or incompletely understood [20]. However, combining all information in a usable format, known as bioinformatics, opens new doors for the future selection of high-production dairy cattle [20,122]. The critical information lies in the subtle changes in gene expression and the cumulative effect that these changes may have [20]. Traditional genetic selection methods in dairy cattle will complement genetic data in the near future [20,123]. The long-term prognosis for genome science is good, but progress will take time. Genetic selection in the genome era will be different because DNA sequence analysis may replace traditional methods for dairy genetic selection [7,13,20,124,125,126,127]. 

The study of microRNAs (miRNAs) remains a challenge. miRNAs are non-coding RNAs that play an essential role in post-transcriptional regulation [128]. *miRNA-148th* is highly expressed and plays a crucial role in epigenetic regulation by decreasing the expression of DNA methyltransferase 1 [103]. Another important miRNA in milk is *miRNA-125b,* which regulates p53 [128]. Therefore, milk-derived miRNAs could be considered potential biomarkers for different mammary gland traits because changes in immunoregulatory miRNAs have been observed in quarters with chronic subclinical mastitis, with *bta-miR-223-3p* being the most upregulated miRNA [129]. Furthermore, recent advances report an essential role of exosomal miRNAs in cell signaling pathways regulating mammary gland immune response in ruminants [130]. In the immediate future, understanding miRNAs will enable their application as genomic markers in dairy cattle as a screening tool for udder health. For example, the miR-200 family of miRNAs plays an essential role in inhibiting the growth and progression of future mammary tumors. Thus, the miR-200 family could also offer a preventive strategy. In addition, increased miR-200 expression in late gestation and early lactation could be a determinant for the development of mammary gland alveoli or the regulation of milk production [131]. The regulatory role of miRNAs in response to most mastitis-causing pathogens is not yet well understood. However, discovering the function of miRNAs and their post-transcriptional regulation would aid in understanding the response of the bovine mammary gland to pathogens and improve lactation efficiency, milk quality, and longevity [132,133].

Besides genomic selection through genetic markers to predict an animal’s breeding value for udder health and milk productivity, other emerging technologies include automated milking systems [69], which allow for the real-time data collection on milk yield and udder health. Additionally, new imaging technologies, such as ultrasound [134] and thermography [135], can be used to assess udder health and identify potential issues early on. Machine learning methods, such as neural networks and decision trees, have shown promising results in predicting udder health status based on somatic cell counts. These methods can help identify cows at risk of mastitis and enable earlier interventions. Artificial intelligence, including deep learning algorithms, will likely be explored in the context of udder health prediction [67]. As data collection and analysis technologies continue to improve, it is likely that machine learning and artificial intelligence will play an increasingly important role in predicting and managing udder health in dairy cattle. In the near future, it is likely that these technologies will continue to advance and become more widely used in the dairy industry, potentially leading to more accurate and efficient selection for udder-related traits.

## 7. Conclusions

Implementing genomic, genotypic, and phenotypic udder evaluation criteria for dairy cattle selection provides solid predictions for improving udder health, milk productivity, and longevity characteristics, thus generating more sustainable genetic lines. The estimation criteria of the different udder-derived traits regarding functionality and conformation should be considered as a whole. Furthermore, the association of both genomic and phenotypic traits significantly improves the accuracy of the prediction values. Thus, udder-derived estimation criteria should be based on different genomic and phenotypic databases to make predictions more reliable. Selection criteria based on genomic and phenotypic udder-derived traits provide a better understanding of the cattle’s health, milk yield and quality, welfare, and herd life. This insight becomes highly relevant as estimation criteria derived from genomic and phenotypic data repositories regarding udder-derived traits point the way for selecting future herds and accelerating the process of genetic improvement in dairy cattle.

## Data Availability

Data sharing is not applicable to this article.
